# In utero estrogenic endocrine disruption alters the stroma to increase extracellular matrix density and mammary gland stiffness

**DOI:** 10.1186/s13058-020-01275-w

**Published:** 2020-05-05

**Authors:** Clarissa Wormsbaecher, Andrea R. Hindman, Alex Avendano, Marcos Cortes-Medina, Caitlin E. Jones, Andrew Bushman, Lotanna Onua, Claire E. Kovalchin, Alina R. Murphy, Hannah L. Helber, Ali Shapiro, Kyle Voytovitch, Xingyan Kuang, Renan Aguilar-Valenzuela, Jennifer L. Leight, Jonathan W. Song, Craig J. Burd

**Affiliations:** 1 Department of Molecular Genetics, The Ohio State University, 920 Biomedical Research Tower, 460 W. 12th Ave., Columbus, OH 43210 USA; 2 The Ohio State University Comprehensive Cancer Center, Columbus, OH USA; 3 Department of Biomedical Engineering, The Ohio State University, Columbus, OH USA; 4 Department of Chemical and Biomedical Engineering, The Ohio State University, Columbus, OH USA; 5 Department of Mechanical and Aerospace Engineering, The Ohio State University, Columbus, OH USA

**Keywords:** Bisphenol A, Bisphenol S, Diethylstilbestrol, Endocrine disruptors, Breast cancer, Collagen, Mammary gland stiffness, Extracellular matrix

## Abstract

**Background:**

In utero endocrine disruption is linked to increased risk of breast cancer later in life. Despite numerous studies establishing this linkage, the long-term molecular changes that predispose mammary cells to carcinogenic transformation are unknown. Herein, we investigated how endocrine disrupting compounds (EDCs) drive changes within the stroma that can contribute to breast cancer susceptibility.

**Methods:**

We utilized bisphenol A (BPA) as a model of estrogenic endocrine disruption to analyze the long-term consequences in the stroma. Deregulated genes were identified by RNA-seq transcriptional profiling of adult primary fibroblasts, isolated from female mice exposed to in utero BPA. Collagen staining, collagen imaging techniques, and permeability assays were used to characterize changes to the extracellular matrix. Finally, gland stiffness tests were performed on exposed and control mammary glands.

**Results:**

We identified significant transcriptional deregulation of adult fibroblasts exposed to in utero BPA. Deregulated genes were associated with cancer pathways and specifically extracellular matrix composition. Multiple collagen genes were more highly expressed in the BPA-exposed fibroblasts resulting in increased collagen deposition in the adult mammary gland. This transcriptional reprogramming of BPA-exposed fibroblasts generates a less permeable extracellular matrix and a stiffer mammary gland. These phenotypes were only observed in adult 12-week-old, but not 4-week-old, mice. Additionally, diethylstilbestrol, known to increase breast cancer risk in humans, also increases gland stiffness similar to BPA, while bisphenol S does not.

**Conclusions:**

As breast stiffness, extracellular matrix density, and collagen deposition have been directly linked to breast cancer risk, these data mechanistically connect EDC exposures to molecular alterations associated with increased disease susceptibility. These alterations develop over time and thus contribute to cancer risk in adulthood.

## Background

In utero exposure to estrogenic endocrine disruptors (EDCs) is associated with increased risk of breast cancer. Women exposed in the womb to diethylstilbestrol (DES) have twice the risk of developing the disease after the age of 40 [1, 2]. While in utero exposures to other endocrine disruptors are hard to quantify in the human population, animal models have demonstrated that in utero exposures to many EDCs can also increase the risk of tumorigenesis. One of the most prevalent industrial EDCs is bisphenol A (BPA), which is used in plastics, canned food linings, thermal paper receipts, and other consumer products. Its abundant use has led to significant human exposures and is detected in human serum, urine, amniotic fluid, and fetal serum [3, 4]. The ability of BPA to act as an estrogen has raised concerns over these exposures, leading to its replacement in various commercial products. These BPA replacements have varying levels of estrogenic activity with compounds such as bisphenol AF having higher estrogenic activity than BPA and bisphenol S (BPS) having lower estrogenic activity [5–7]. As the number of EDCs expands, understanding the critical properties and mechanisms driving risk becomes more imperative.

Rodent models demonstrate that early life exposures to estrogenic EDCs have long-term consequences on mammary gland development and promote tumorigenesis. In utero BPA exposures induce preneoplastic lesions and hyperplasia in rat mammary glands [8, 9]. Mice exposed to BPA in utero develop tumors more quickly and more often when challenged with a chemical carcinogen [10]. Taken together, these rodent models show that early life BPA exposure increases the risk of mammary gland carcinogenesis. While BPA has been extensively studied for both developmental defects and cancer-associated phenotypes in rodent models, there is much less data on the BPA alternatives. However, BPS has been shown to drive many of the same developmental abnormalities in the mammary gland as seen in BPA-exposed animals [11]. While no study has demonstrated cancer formation in BPS-exposed mice, a small number of mice did have mammary neoplasias in one study [12]. These data suggest that compounds such as BPS are also a potential risk, yet no defined mechanism by which these compounds initiate oncogenesis has been identified. It is unclear whether the estrogenic properties of these EDCs are responsible for the increased cancer risk and which molecular alterations within the gland predispose the mammary cells to carcinogenic transformation. Without this knowledge, it is difficult to test and identify potentially hazardous compounds and account for latent effects following early life exposures.

As many of the EDCs that alter mammary gland development are estrogenic, the ability of these chemicals to stimulate the estrogen receptor is used as the fundamental characteristic to identify them as potentially harmful compounds. Attempts to link the estrogenic action of BPA to the phenotypic changes in the mammary gland implicate the developing mesenchyme as a potential cellular target of EDC action [13]. The developing murine mammary bud is completely surrounded by estrogen receptor (ER) expressing mesenchymal cells from embryonic day 12.5 to 15.5 [14]. This period during development appears to be the time point most susceptible to BPA action [14]. Furthermore, transcriptome profiling of the perinatal stroma and epithelium of the developing mammary gland at the time of exposure suggests that BPA is acting as an estrogen through the mesenchymal tissue [15]. This transcriptional profiling suggests that the extracellular matrix (ECM) is differentially regulated by BPA exposures [15, 16]. As growth, survival, and differentiation of the epithelium are dependent upon signals from the stroma [17], these alterations can influence carcinogenic transformation later in life. To this end, we aimed to analyze the long-term changes within the fibroblast population that persist into adulthood when carcinogenic transformation occurs. In this study, we characterize stromal alterations that are only evident in the adult female mouse and are known risk factors for human breast cancer.

## Methods

### Animals

Animal experiments were performed in compliance with protocols approved by The Ohio State University Institutional Animal Care and Use Committee (IACUC, Protocol #2013A00000030) and in accordance with the accepted standard of humane animal care. CD1 mice (Charles Rivers, Wilmington, MA, USA) were maintained in polysulfone cages and fed a diet containing minimal levels of phytoestrogen (Harlan2019X). BPA dissolved in sesame oil was administered to pregnant mice via intraperitoneal (IP) injection as previously described [14], unless otherwise stated in the text. Treatment with 25 μg/kg · bodyweight BPA (≥ 99%), 100 μg/kg · bodyweight DES (99% (HPLC)), 25 μg/kg · bodyweight BPS (analytical standard), or equal volume sesame oil control occurred daily from embryonic day 9.5 through 18.5 (E9.5–18.5). Specific lots of EDCs were tested for estrogenic activity via luciferase reporter assay (Supplemental Figure 1). Resulting female offspring were harvested at 4 and 12–14 weeks of age for analyses as indicated.

### Luciferase reporter

Luciferase reporter assay was performed as previously described [18], with minor modifications. Cells were treated with 1 nM, 10 nM, 100 nM, or 1 μM estradiol (E2), BPA, BPS, DES, or ethanol (EtOH) control for 24 h before analysis.

### Fibroblast RNA isolation and sequencing

Mammary fibroblast cells were isolated from adult mice, as previously described [19]. Briefly, in utero exposed female mice were harvested between 12 and 14 weeks of age. The fourth and fifth inguinal mammary glands were excised from up to 4 litters totaling between 12 and 20 mice, with lymph nodes excluded, and mechanically minced to produce a fine, semi-liquid slurry. The slurry was further digested in a collagenase and trypsin mixture on a platform shaker (150 rpm at 37 °C for 1 h). The mixture was centrifuged (350×*g* for 3 min) to collect the epithelial organoids, stromal cells, fibroblasts, and red blood cells in the pellet. This pellet was rinsed in red blood cell lysis buffer (Sigma-Millipore) two times and resuspended in Dulbecco’s Modified Eagle’s Medium (DMEM) containing 10% fetal bovine serum (FBS). Cells were plated onto a cell culture dish for 1 h to separate the epithelial organoids from fibroblasts. Adherent fibroblasts were maintained at 37 °C/5% CO_2_/5% O_2_ until 80% confluent. Plates were washed with 1× phosphate-buffered saline (PBS) and DNA and RNA harvested using the ZR-Duet DNA/RNA mini prep kit (Zymo Research).

### Fibroblast staining and flow cytometry

Fibroblasts were collected by trypsinization, washed with 3% bovine serum albumin (BSA) in PBS, and then incubated with Ghost Dye Red 780 Viability Dye stain (1:1000, Tonbo Bioscience, 13-0865-T100) for 10 min at room temperature. Cells were washed with 3% BSA in PBS, fixed with 0.1% paraformaldehyde for 15 min at room temperature, washed again with 3% BSA in PBS, and permeabilized with 2% saponin for 15 min at room temperature. Cells were then incubated with Fc blocking anti-mouse CD16/CD32 antibody (1:100, clone 2.4G2; Tonbo Bioscience, 70-0161-M001) for 10 min. After an additional 3% BSA in PBS wash, cells were labeled with primary antibodies to fibroblast-specific protein 1 (FSP1, Millipore, 07-2274), fibroblast activation protein α (FAP, R&D Systems, MAB9727), α smooth muscle actin (αSMA, Millipore, A2547), platelet-derived growth factor receptor α (PDFGRα, Cell Signaling, 3174), platelet-derived growth factor receptor β (PDGFRβ, Cell Signaling, 3169), Vimentin (Cell Signaling, 5741), or secreted protein acidic and rich in cysteine (SPARC, Cell Signaling, 8725) for 30 min at 4 °C. Again, cells were washed with 3% BSA in PBS and then followed by incubation with appropriate fluorescent secondary antibodies for 30 min at 4 °C. Cells were washed with 3% BSA in PBS three times prior to analysis on a flow cytometer (BD LSRFortessa).

### Differential gene expression analysis

Sequencing reads were mapped to the MM10 genome using the STAR read aligner [20], and transcript read counts quantified using featureCounts [21]. Low expression genes with less than 10 reads across all samples were filtered out of the dataset, and differentially expressed genes identified using DESeq2 criteria (*p* ≤ 0.05, FDR 5%) [22]. RNA-seq datasets are available through GEO series accession number: GSE136062. Statistically significant gene lists were subjected to Ingenuity Pathway Analysis (Qiagen Bioinformatics) and the ToppGene Suite [23].

### RT-PCR

Fibroblast RNA was converted to cDNA using the iScript Select cDNA Synthesis Kit (BioRad, 170-8897). Relative gene expression was determined by real-time qPCR using SYBR Green (BioRad) with the primers listed in Supplemental File 1.

### Picrosirius red staining

The fourth inguinal mammary glands were harvested at either 4 weeks or 12 weeks of age, for oil and BPA treatments. Mice were staged in the estrous cycle prior to harvest using vaginal cytology [24–26] and harvested in the estrus stage. Estrus stage was confirmed through hematoxylin and eosin analysis of the dissected uterus, ovaries, and vagina from each mouse [24, 25]. Glands were fixed in 10% neutral buffered formalin for 48 h, transferred to 70% ethanol, and paraffin embedded. The paraffin-embedded mammary glands were cut in 4 μm sections for picrosirius red staining. A picrosirius red staining kit (Abcam, ab150681) was used to visualize collagen fibers.

Images were taken under 4× magnification with a light microscope (Nikon Eclipse 50i microscope) equipped with a camera (Axiocam color, Zeiss) and Zeiss Zen Pro software. For 4-week-old mice, one image was taken per gland section, between the lymph node and nipple. For 12-week-old mice, two images were taken adjacent to the lymph node. Images were de-identified, cropped to identical size, and portions of the lymph node cut from the image. Images were then blindly scored in ImageJ by measuring the total area of each image and then the area with signal above a standardized threshold on the red channel. Data represents the percent area above the threshold compared to total area of the image.

### Hydroxyproline assay

Hydroxyproline levels were determined using the Sigma Hydroxyproline Assay Kit (Sigma-Aldrich/Millipore, MAK008) using 10 mg pieces of mammary gland excised from the fourth inguinal mammary gland from 12-week-old mice treated in utero with either BPA or oil.

### Second harmonic generation imaging and collagen fiber analysis

Collagen fibers were imaged in picrosirius red-stained tissue sections from the fourth inguinal mammary glands of 12-week-old mice using an Olympus FV1000 MPE microscope equipped with a × 25 XLPlan objective (N.A. 1.05) and a Mai Tai DeepSee Ti:Sapphire Laser (Spectra-Physics, Newport Corp.) at a 950 nm wavelength. Images were taken through the depth of the sample in 4 μm sections, focusing on mammary ducts and the periductal area. A maximum intensity projection of these images was taken, and CT-Fire software (v1.3; https://loci.wisc.edu/software/ctfire) was used to analyze the collagen fibers in each image. Three glands from oil-treated mice and 4 glands from BPA-treated mice were imaged, and 3 images were taken per tissue section. Overall, 467 collagen fibers in oil-treated mice and 1256 collagen fibers in BPA-treated mice were analyzed.

### Hydraulic permeability assay

The measurement of ECM hydraulic permeability was performed with in utero exposed fibroblasts. Re-organization of commercially available collagen I was performed as previously described [27]. Briefly, neutralized (NaOH) acidic rat tail type I collagen (Corning) solutions were prepared at 6 mg/mL in media for the following separate experimental conditions: (1) containing in utero oil-treated fibroblasts, (2) containing in utero BPA-treated fibroblasts (1800 cells/μL), and (3) no added fibroblasts for acellular conditions. Polydimethylsiloxane microfluidic channels were pre-coated with 100 μg/mL fibronectin prior to collagen mixture injection, as previously determined to be optimum. A 5 mg/mL solution of tetramethylrhodamine conjugated BSA (Thermo Fisher, A23016) in 1× PBS was used to monitor flow through the channel.

### Mammary stiffness assay

The elastic modulus of mammary glands was determined using an unconfined compression protocol, as previously described [28–30]. Following in utero exposures, the fourth inguinal mammary glands were harvested from mice at either 4 weeks or 12 weeks of age, for each treatment group. Using a biopsy punch, circular sections approximately 4 mm in diameter with a thickness of 1 mm were excised from the fourth gland, avoiding the lymph node and excising sections towards the leading edge of the mammary gland. Sections were loaded on a mechanical testing system (Electroforce 5500, TA Instruments, Eden Prairie, MN) and compressed at a strain rate of 0.5 mm/min to a final strain of 30%. The modulus was estimated by calculating the slope of the stress-strain curve. A minimum of three sections per mouse were analyzed and averaged per data point.

### Statistical analyses

An unpaired *t* test with Welch’s correction determined statistical significance between oil- and BPA-treated mice for qPCR analyses of collagen genes, picrosirius red analyses, hydroxyproline analyses, the number of fibers per duct from SHG measurements, and stiffness analyses. For SHG measurements with histograms of fiber length and fiber width, a two-sample Kolmogorov-Smirnov test was used. A one-way ANOVA with Dunnett’s multiple comparisons was used to determine significance for hydraulic permeability data, luciferase assay, and the mammary gland stiffness analyses that compared oil, BPS, and DES.

## Results

### In utero BPA alters the transcriptome of fibroblasts in adult female mice

**Fig. 1:**
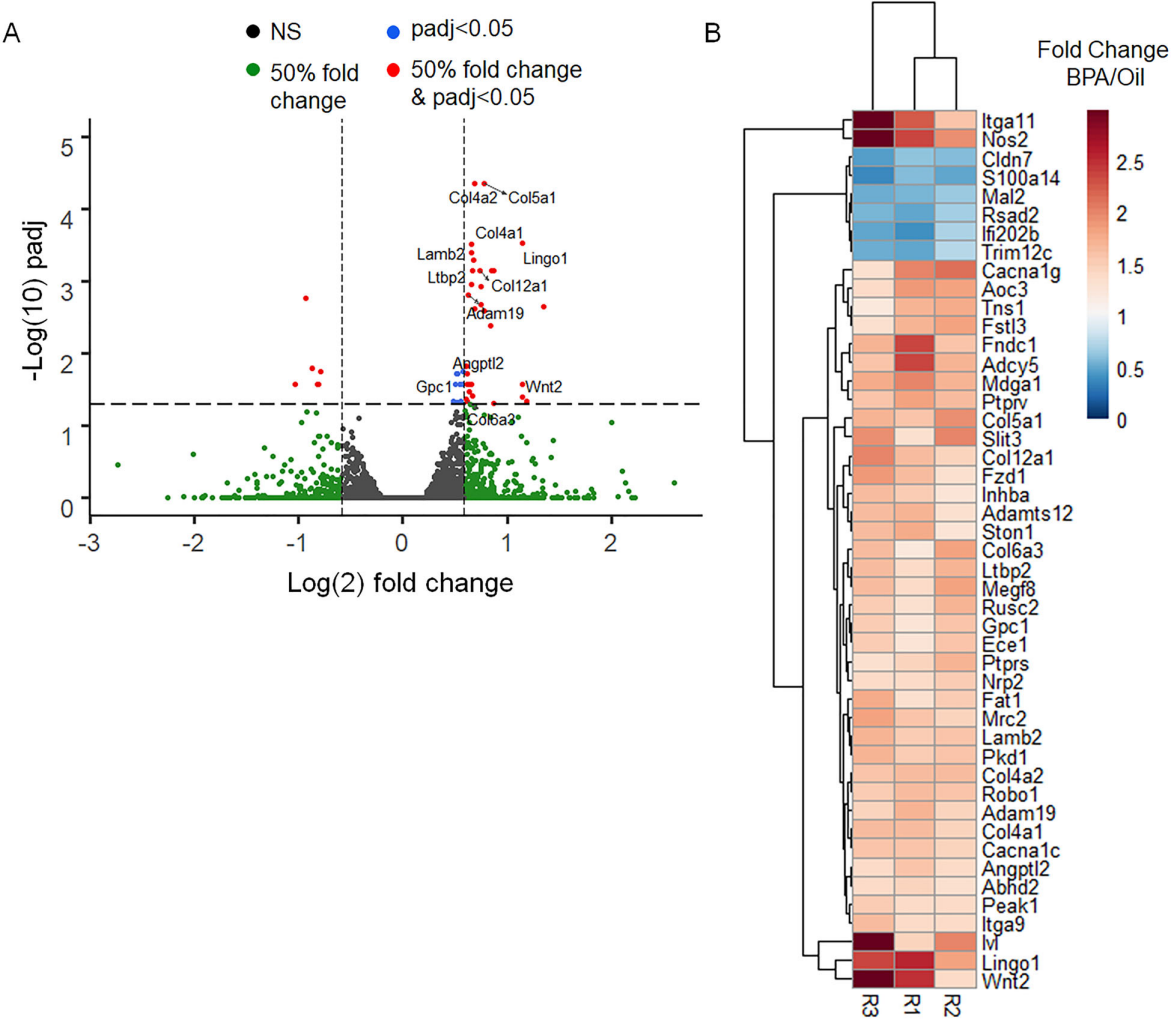
In utero BPA exposure drives long-term changes in mammary fibroblast gene expression. Fibroblasts were isolated from the mammary glands of 12-week-old female mice exposed in utero to 25 μg/kg · bodyweight BPA or oil control from E9.5 through E18.5. RNA from the fibroblasts was sequenced and analyzed for differential gene expression. **a** A volcano plot is depicted of all analyzed genes with cutoffs for fold change, *p* value, and *p* adjusted (*p*adj) value depicted. **b** A heatmap of the 47 genes with a *p*adj value ≤ 0.05 is depicted illustrating the fold change between BPA- and oil-exposed fibroblasts in each of the three cohorts of mice used for the transcriptome analysis. These analyses were done in 3 separate cohorts of mice treated with either oil or BPA in utero

Several studies have implicated the mesenchymal cells surrounding the developmental mammary bud as a target of in utero BPA action [14–16]. In addition, the stroma plays a key role in mammary gland development and cancer risk through paracrine signaling and ECM interactions [17]. To this end, we attempted to identify changes within the stroma that occur after in utero BPA exposure that may increase cancer risk. Pregnant CD1 mice were exposed to 25 μg/kg · bodyweight BPA or oil control from E9.5 through E18.5. We have previously shown this dose results in amniotic BPA levels comparable to reported human amniotic levels [14]. Following birth, mice were aged 12–14 weeks to allow for complete epithelial ductal elongation within the mammary gland and to approximate an adult time point when increased carcinogenic risk is suspected. Fibroblasts isolated from mammary glands were validated by flow cytometry for expression of the markers fibroblast-specific protein 1 (FSP1, gene name S100A4) and smooth muscle actin (αSMA) (Supplemental Figure 2A). We did not observe a BPA-induced change in fibroblast subpopulations as measured by the specific markers, FAP, SPARC, Vimentin, PDFGRα, and PDGFRβ (Supplemental Figure 2B) [31]. RNA was isolated from mammary fibroblasts and subjected to whole genome transcriptome profiling. Mice exposed to in utero BPA had 489 differentially regulated genes (*p* ≤ 0.05, ≥ 50% change) and 47 statistically significant (*p*adj < 0.05) altered transcripts when accounting for false discovery (Fig. 1a, Supplemental File 2). Interestingly, nearly all (41 of 47) of the altered genes had increased expression in the BPA-exposed mice (Fig. 1). Gene expression changes of these 47 genes for our 3 cohorts of mice were consistent, although very few genes showed greater than a 2-fold change in expression (Fig. 1b. Ingenuity Pathway Analysis of these 47 genes identified cancer, including breast, as the disease most associated with these alterations (Table 1). In addition, genes associated with the ECM and collagen regulation were the molecular functions and cellular components most enriched (Table 1).

**Table 1:** Molecular functions, cellular components, pathways, and diseases enriched in genes deregulated in fibroblasts following in utero BPA exposure

	*p*-value
**Molecular Function**	
Extracellular matrix structural constituent conferring tensile strength	4.02E-08
Extracellular matrix structural constituent	1.63E-07
Voltage-gated calcium channel activity involved in AV node cell action potential	1.54E-05
Collagen binding	3.20E-05
**Cellular Component**	
Extracellular matrix	4.87E-09
Collagen-containing extracellular matrix	5.35E-09
Extracellular matrix component	1.41E-07
**Pathway**	
Extracellular matrix and extracellular matrix-associated proteins	8.92E-10
Extracellular matrix organization	3.09E-08
Integrin signaling pathway	5.81E-08
NCAM1 interactions	1.21E-07
Core extracellular matrix including ECM glycoproteins, collagens and proteoglycans	2.07E-07
ECM-receptor interaction	2.54E-07
Genes encoding collagen proteins	2.97E-07
Collagen chain trimerization	4.17E-07
Collagen biosynthesis and modifying enzymes	3.11E-06
**Disease**	
Colorectal cancer	9.22E-10
Breast or pancreatic cancer	9.99E-10
Breast or ovarian cancer	1.39E-09
Development of vasculature	1.73E-09
Angiogenesis	1.89E-09
Breast or gynecological cancer	6.34E-09

**Fig. 2:**
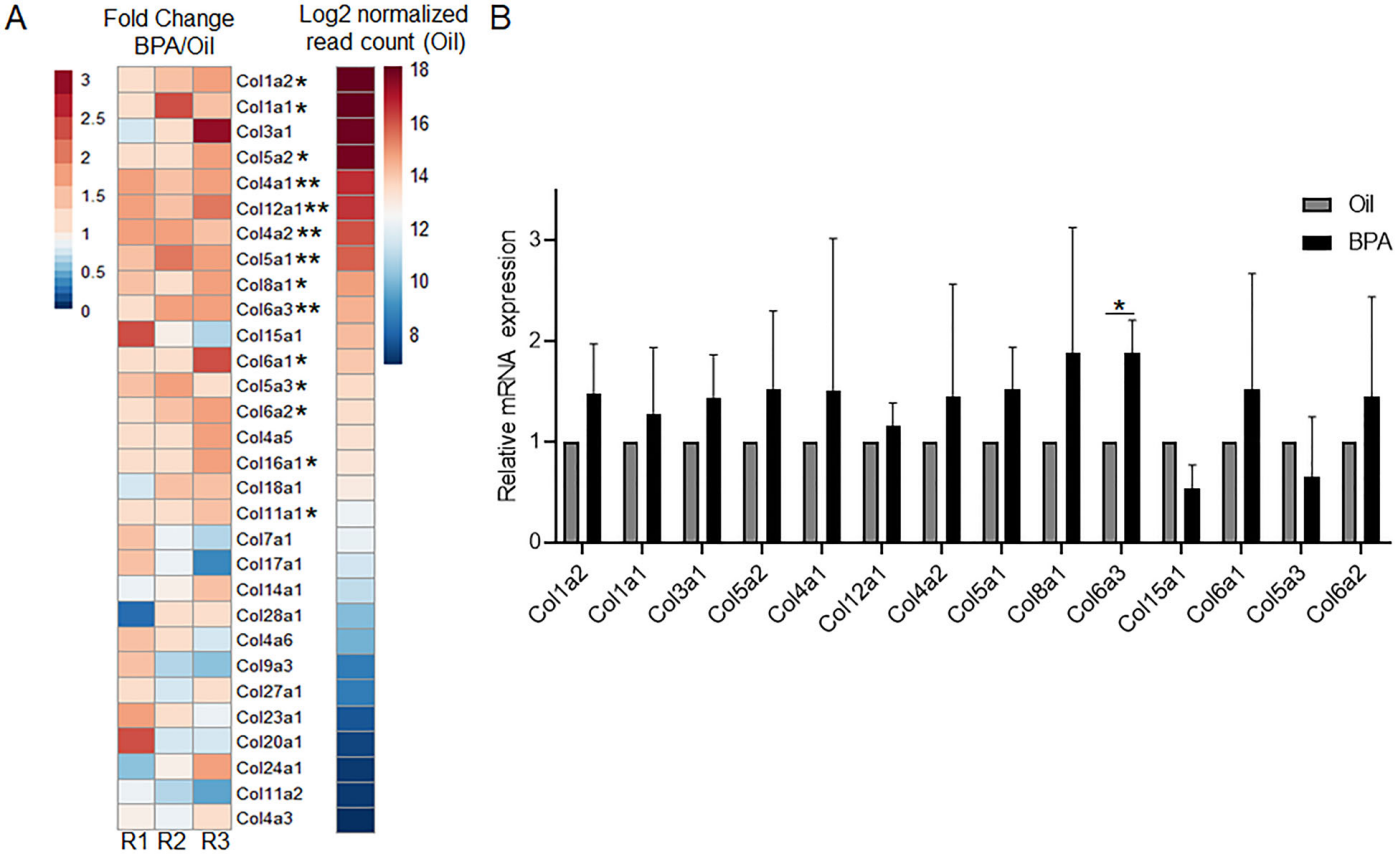
Collagen genes are more highly expressed in BPA-exposed mammary glands. **a** A heatmap showing the RNA expression of the most abundantly expressed collagen genes is depicted. Collagen genes are ordered from top to bottom based upon the average normalized read counts in the control samples. **padj < 0.05; *p < 0.05 **b** Collagen genes that had increased expression in our RNA-seq dataset were validated on an additional 3 cohorts of mice using qPCR. Average normalized mean expression ± SD is graphed. *p < 0.05

Changes in collagen deposition have been proposed as a risk factor for breast cancer [32, 33], so we investigated the collagen family of genes in more detail. A total of 15 collagen genes demonstrated a change in expression (*p* ≤ 0.05) in BPA-exposed mice. In fact, increased collagen expression in BPA-exposed mice was observed in our 3 cohorts (R1, R2, R3) especially amongst the most highly expressed collagen genes (Fig. 2a). We thus validated these changes amongst an additional 3 cohorts of 12–14-week-old mice whose mothers were exposed to BPA or oil via oral gavage, as opposed to IP injection. In these additional samples, we again see a general increase of 20–50% expression across the most highly expressed collagen genes when exposed in utero to BPA (Fig. 2b). In line with previous studies, the route of exposure does not affect this phenotype [34].

### BPA exposure increases collagen deposition in adult female mice

**Fig. 3:**
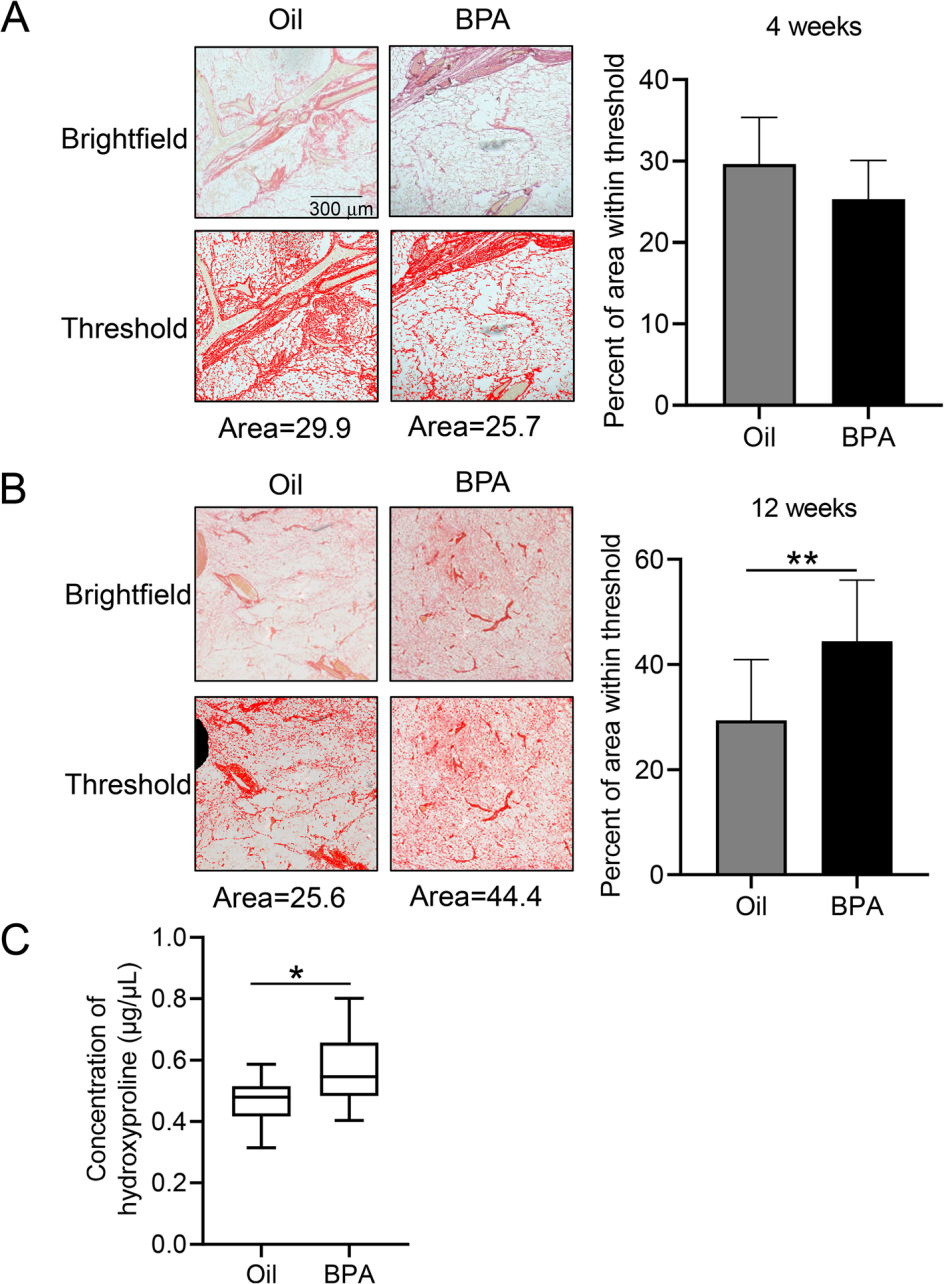
Collagen gene expression is altered in mice exposed in utero to BPA. **a** Mammary glands from 4-week-old mice exposed to BPA or oil in utero were stained with picrosirius red to visualize collagen. Total collagen staining was quantified by the area of red staining in images above the threshold (right graph) and graphed as mean ± SD. A representative brightfield image and threshold image used for quantification are depicted. Area in red of threshold image represents the area quantified. Selected images represent the data point closest to the mean for that sample set. *N* = 7 for oil and *N* = 5 for BPA, from a minimum of 3 separate litters. **b** Mammary glands from 12-week-old mice quantified and graphed as mean ± SD as in **a** for total area of the mammary gland stained for collagen. *N* = 11 for oil and *N* = 13 for BPA, from a minimum of 3 separate litters. **c** Hydroxyproline concentration was measured in mammary glands from 12-week-old mice exposed to BPA or oil in utero*. N* = 10 for oil and *N* = 9 for BPA, from a minimum of 3 separate litters. *p < 0.05; **p < 0.01

In order to determine if these transcriptional changes result in increased collagen deposition in adult mice, we stained mammary glands of BPA-exposed and control mice with picrosirius red. We performed these analyses early in mammary development at 4 weeks of age and again after complete ductal elongation at 12 weeks of age. At 4 weeks of age, we see no change in collagen expression between BPA and control mice (Fig. 3a, Supplemental Figure 3). However, at 12 weeks of age, BPA-exposed mice have significantly more area of the mammary gland stained for collagen deposition (Fig. 3b, Supplemental Figure 3). In order to validate the increases in total collagen deposition in 12-week-old mice, we measured the amount of hydroxyproline present in 10 mg pieces of mammary tissue. Similar to our quantification of picrosirius red staining, an increase in hydroxyproline is observed in mice that were exposed to BPA in utero (Fig. 3c).

### BPA exposure increases the number of collagen fibers and alters the structure of the collagen fibers in mammary glands of adult female mice

**Fig. 4:**
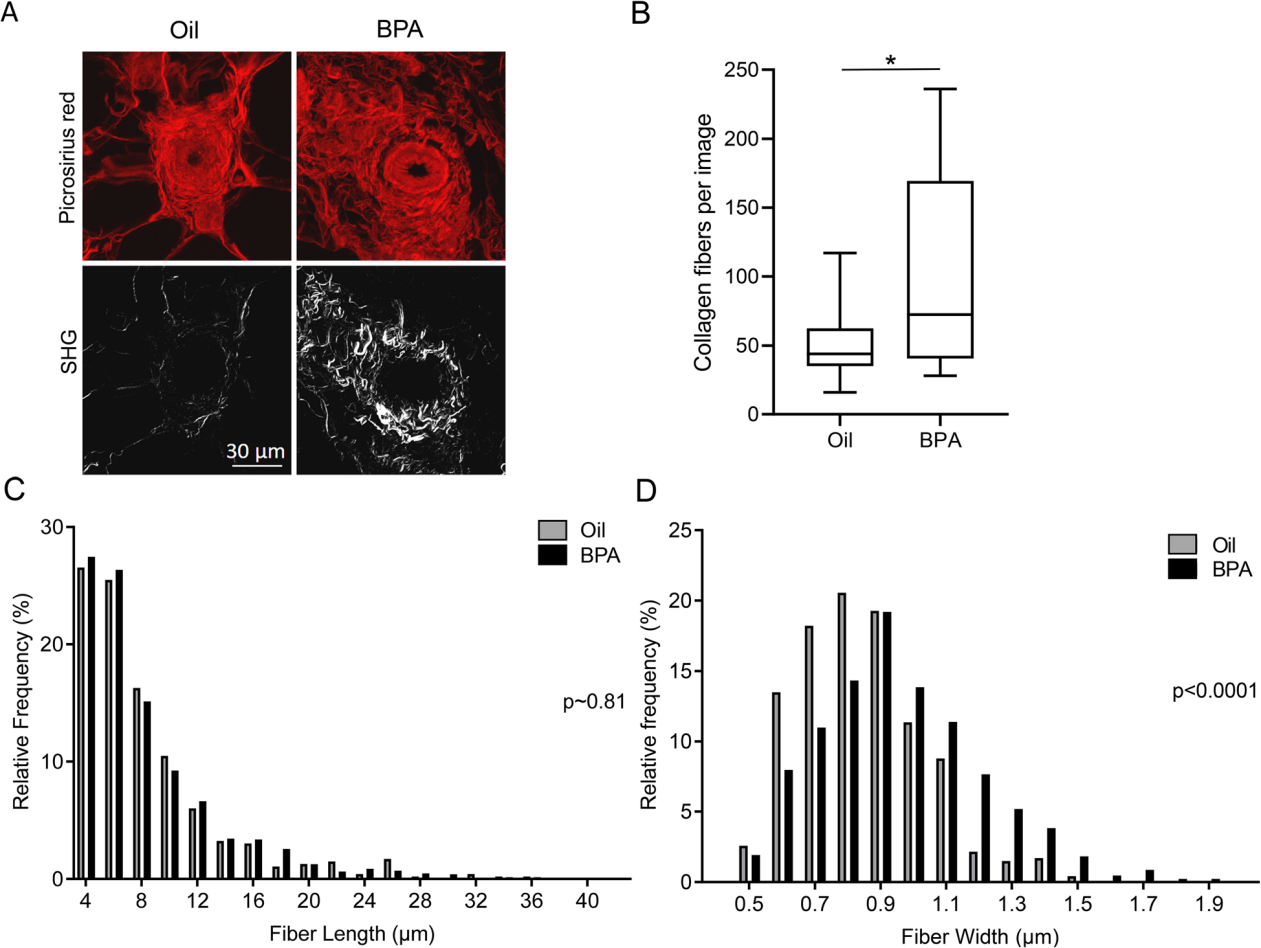
BPA exposure increases the number of collagen fibers and alters the structure of the collagen fibers in mammary glands of adult female mice. **a** Representative images of second harmonic generation imaging and corresponding picrosirius red staining surrounding mammary ducts in 12-week-old mice. **b** Quantification of the number of fibers measured by second harmonic generation imaging is depicted. **c** Histogram of collagen fiber length, quantified using the CT-Fire software. Bin size = 2 μm. **d** Histogram of collagen fiber width, quantified using the CT-Fire software. Bin size = 0.1 μm. Three glands from oil-treated mice and 4 glands from BPA-treated mice were imaged, and 3 images were taken per tissue section. Overall, 467 collagen fibers in oil-treated mice and 1256 collagen fibers in BPA-treated mice were analyzed, from a minimum of 3 separate litters. *p < 0.05

To further characterize collagen fibers in the mammary glands, tissue sections from 12-week-old mice were imaged using second harmonic generation microscopy (SHG). Mammary ducts were identified using the picrosirius red staining, and the periductal area was imaged using SHG (Fig. 4a). While picrosirius red stains a variety of collagens, SHG primarily images mature collagen I fibers. There was a significant increase in the number of collagen type I fibers in glands from BPA-treated mice (Fig. 4b). The length and width of these fibers were quantified, and the distributions of length and width were plotted as histograms. Although there was no significant change in the length of these fibers (Fig. 4c), the distribution of collagen fiber width was significantly shifted to the right, indicating an increase in fiber width in BPA-treated mice (Fig. 4d). This increase in both collagen I fiber number and fiber diameter contributes to an expansion in overall collagen I content of the gland with BPA treatment.

### In utero BPA decreases collagen matrix hydraulic permeability and increases mammary gland stiffness

**Fig. 5:**
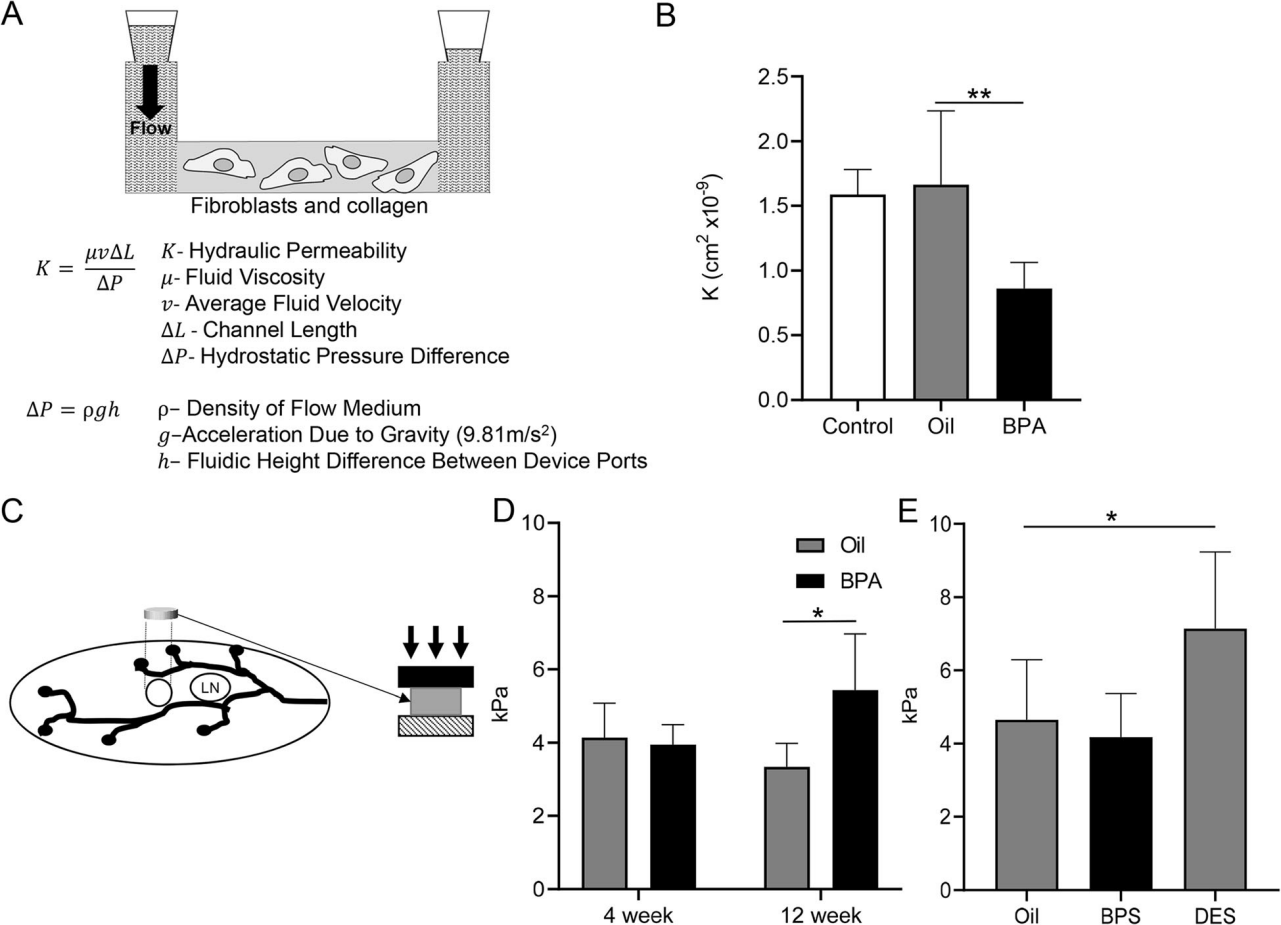
BPA decreases collagen matrix permeability and increases mammary gland stiffness. **a** An in vitro assay was utilized to measure fibroblast organization of a collagen matrix. A PDMS microfluidic channel was pre-coated with fibronectin and then, fibroblasts isolated from 12-week-old mice were plated with collagen. After 48 h of fibroblast-mediated remodeling of the collagen matrix, a fluorescent liquid was pumped through the channel. Measurement of the pressure and flow velocity was used to calculate the permeability (*K*) of the collagen matrix arranged by the fibroblasts. **b** Mammary fibroblasts were isolated from BPA and control 12-week-old mice and cultured within the channel as depicted in **a**. ECM permeability was measured, and the graph represents the mean ± SD. Control represents the permeability of collagen with no cultured fibroblasts. *N* = 4 for oil and *N* = 6 for BPA. **c** The diagram depicts the methodology to measure mammary gland stiffness. A circular section approximately 4 mm diameter and 1 mm thick was removed adjacent to the lymph node from mammary glands. The amount of applied stress required to compress these disks is measured to determine the stiffness of the gland. **d** Mammary glands from 4- and 12-week-old mice were analyzed as described in **c** for stiffness. Measurements of the elastic modulus are graphed as mean ± SD. For 4-week-old samples, *N* = 4 for oil and *N* = 4 for BPA, from a minimum of three separate litters. For 12-week-old samples, *N* = 3 for oil and *N* = 4 for BPA, from a minimum of three separate litters. **e** A cohort of mice were treated in utero with either 100 μg/kg · bodyweight DES, 25 μg/kg · bodyweight BPS, or oil control from E9.5 through E18.5. At 12 weeks of age, mammary glands were harvested and gland stiffness measured as depicted in **c**. Measurements are graphed as mean ± SD. *N* = 5 for oil, *N* = 11 for BPS, and *N* = 5 for DES. *p < 0.05; **p < 0.01

Collagen expression and breast density are significant risk factors for breast cancer [35–42]. Thus, we aimed to determine if fibroblast-mediated stromal reprogramming is increasing these known risk factors. We first tested the ability of fibroblasts to remodel the ECM in vitro by quantification of hydraulic permeability. Isolated fibroblasts from 12–14-week-old mice exposed to either BPA or oil control in utero were mixed with collagen prior to polymerization in a microfluidic channel. After allowing 48 h for fibroblasts to remodel the collagen matrix, a fluid containing a fluorescent tracer was pumped through the microchannel. By measuring the flow rate through the matrix due to a known pressure gradient, a permeability constant (*K*) can be calculated (Fig. 5a). BPA-exposed mice had decreased collagen permeability as compared to no fibroblasts (control) or oil-exposed fibroblasts (Fig. 5b).

We next aimed to determine if these fibroblast-mediated changes affect mammary stiffness in vivo. We thus adapted established mechanical testing techniques to measure mammary gland stiffness ex vivo (Fig. 5c) [43]. Mice treated in utero with BPA or oil by oral gavage were analyzed for changes in gland stiffness. In line with our transcriptome and in vitro analyses, adult 12-week-old mice have increased mammary gland stiffness as opposed to control mice. Interestingly, 4-week-old mice did not demonstrate any change in mammary gland stiffness between BPA and control mice (Fig. 5d).

As BPA exposure increases ECM collagen content and gland stiffness, which are associated with increased breast cancer risk [39, 44], we wanted to determine if this phenotype is conserved in response to other estrogenic EDCs. To this end, we generated an additional cohort of mice that were exposed in utero to DES, a strong estrogen known to increase breast cancer risk in humans, and BPS, a very weak estrogen used in BPA-free products. Pregnant mice were exposed from E9.5 through E18.5 with 100 μg/kg · bodyweight DES, 25 μg/kg · bodyweight BPS, or oil control via oral gavage. Doses were chosen to be consistent with previous literature in mouse studies or known human exposures [11, 12, 45, 46]. At 12 weeks of age, mice were subjected to our breast stiffness assay. DES-exposed mice demonstrated a significantly increased mammary gland stiffness while the weak estrogen, BPS, was not statistically different from our oil control (Fig. 5e). These data suggest that estrogenicity may be the driving factor of this phenotype and demonstrate that the one EDC known to increase breast cancer risk in humans and is also a strong estrogen (DES) increases mammary gland stiffness associated with the disease.

## Discussion

### BPA induces adult phenotypes associated with human breast cancer risk factors

In this study, we focused our efforts to analyze the changes within the stroma induced by in utero BPA exposures that persist into adulthood when carcinogenic transformation occurs. We identified fibroblast-specific reprogramming of the transcriptome associated with ECM organization. These changes are highlighted by increased collagen deposition and collagen fiber width, decreased hydraulic permeability, and a stiffer mammary gland. Numerous models have demonstrated that altering the ECM alone can contribute to oncogenesis [42]. In fact, mutation of the MMP cleavage site in the Col1a1 gene results in increased collagen content and susceptibility to mammary carcinogenesis [37]. Further, animal and in vitro studies have demonstrated that changes in ECM composition drive a malignant phenotype [42, 47]. Fibroblast-mediated, decreased hydraulic permeability has also been correlated with increased mammary and pancreatic tumor growth in mice [27]. Human data supports these models. Human breast cancer transformation is associated with increases in collagen deposition and fiber thickness [40]. Increased collagen deposition is associated with fibrotic and neoplastic diseases, such as cancer [48]. Decreased hydraulic permeability is known to affect multiple features of tissue, such as interstitial flow and nutrient transport, that may impart mechanical and chemical stresses that promote tumorigenesis [49]. Increased mammary gland stiffness has also been correlated to increased collagen content in the ECM, subsequent tumor promoting functions, and cancer risk [42, 44]. Taken together, our data provides a specific EDC phenotype that has been directly linked to breast cancer risk in humans.

Early life EDC exposure has life-long consequences towards human health. Interestingly, we found that these phenotypes of increased ECM density and gland stiffness, known to be risk factor for breast cancer, were not evident in young mice. These data support the idea that carcinogenic risk progresses throughout life and the impact of these exposures is not necessarily apparent at the time of exposure. In line with this hypothesis is the fact that DES daughters do not display increased breast cancer risk until the women have reached 40 years of age [1, 2].

### BPA alternatives and the importance of estrogenic activity

Identification of new EDCs is mostly driven by the ability to drive a hormone response in various in vitro and in vivo assays. Furthermore, much of the analysis of BPA and its alternatives have used surrogate phenotypes other than tumor formation to implicate cancer risk, such as developmental abnormalities. There is a clear need to understand which EDC-driven phenotypes are actually associated with cancer risk and if those phenotypes are being driven by estrogenic properties of the compounds. Herein, we demonstrate that BPA increases collagen production and mammary gland stiffness, which are known risk factors for breast cancer [37, 40, 42, 44]. In our system, we also show that in utero exposure to the strong estrogen, DES, also increases gland stiffness in the adult animal, while no effect is seen following in utero exposure to the weaker estrogen, BPS. While our study is limited by a single dosage, these data provide impetus to specifically test the link between estrogenic activity and mammary gland stiffness. While we provide evidence that estrogenic EDC exposure can increase these known risk factors for breast cancer, further studies are needed to specifically test the relative contribution of this phenotype to cancer risk in the context of other EDC-driven phenotypes.

## Conclusions

Herein, we identified long-term reprogramming events within the mammary fibroblast population associated with in utero EDC exposures. These events lead to increased collagen production resulting in a stiffer mammary gland. As mammary gland stiffness is associated with human breast cancer risk [44], these data provide a mechanistic link between early life endocrine disruption and adult disease susceptibility.

## Supplementary information

**Additional file 1.**

**Additional file 2.**

**Additional file 3.**

## Data Availability

The RNA-seq data is available through GEO under the series accession number: GSE136062.

## Abbreviations

BPA: Bisphenol A
BPS: Bisphenol S
DES: Diethylstilbestrol
EDC: Endocrine disrupting compound
ECM: Extracellular matrix
ER: Estrogen receptor
