# Supplementary material for: In utero estrogenic endocrine disruption alters the stroma to increase extracellular matrix density and mammary gland stiffness
Source: Breast Cancer Res. 2020 May 5;22:41. doi: 10.1186/s13058-020-01275-w (PMC7201668; doi:10.1186/s13058-020-01275-w)
Supplement: Supplementary file 3 — Additional file 3. [file 13058_2020_1275_MOESM3_ESM.pdf]

Supplemental Figure Legends

Supplemental Figure 1- EDCs utilized in these studies display estrogenic activity. Specific lots of each EDC used in these studies were tested for estrogenic activity by reporter assay. Luciferase activity was measured after 24 hours with either 1 nM, 10 nM, 100 nM, or 1 μM of E2, BPA, BPS,or DES. The data is graphed as mean ± SD. Data represents three technical replicates.* represents $p<0.05$ and *** represents $p<0.0001$.

Supplemental Figure 2- BPA does not alter the relative proportions of fibroblast subpopulations. A. Enrichment of the fibroblasts isolated for these studies was validated by flow cytometry using antibodies labeling the expression of FSP1 and αSMA. Greater than 97% of cells stain positive for FSP1 and αSMA. **B.** Fibroblasts isolated from mammary glands were subjected to flow cytometry using antibodies to detect markers of specific fibroblast subpopulations. Percent positivity of cells for each marker is indicated for each sample in the figure, with the control samples displaying background percent. Data represents 1 biological replicate.

Supplemental Figure 3- Increased collagen deposition in BPA exposed mammary glands in adult female mice. Data represent magnified images of picrosirius red staining under brightfield light from Figure 3 from 4 week old mice (**A**) and 12 week old mice (**B**) exposed to BPA or oil *in utero*.

A

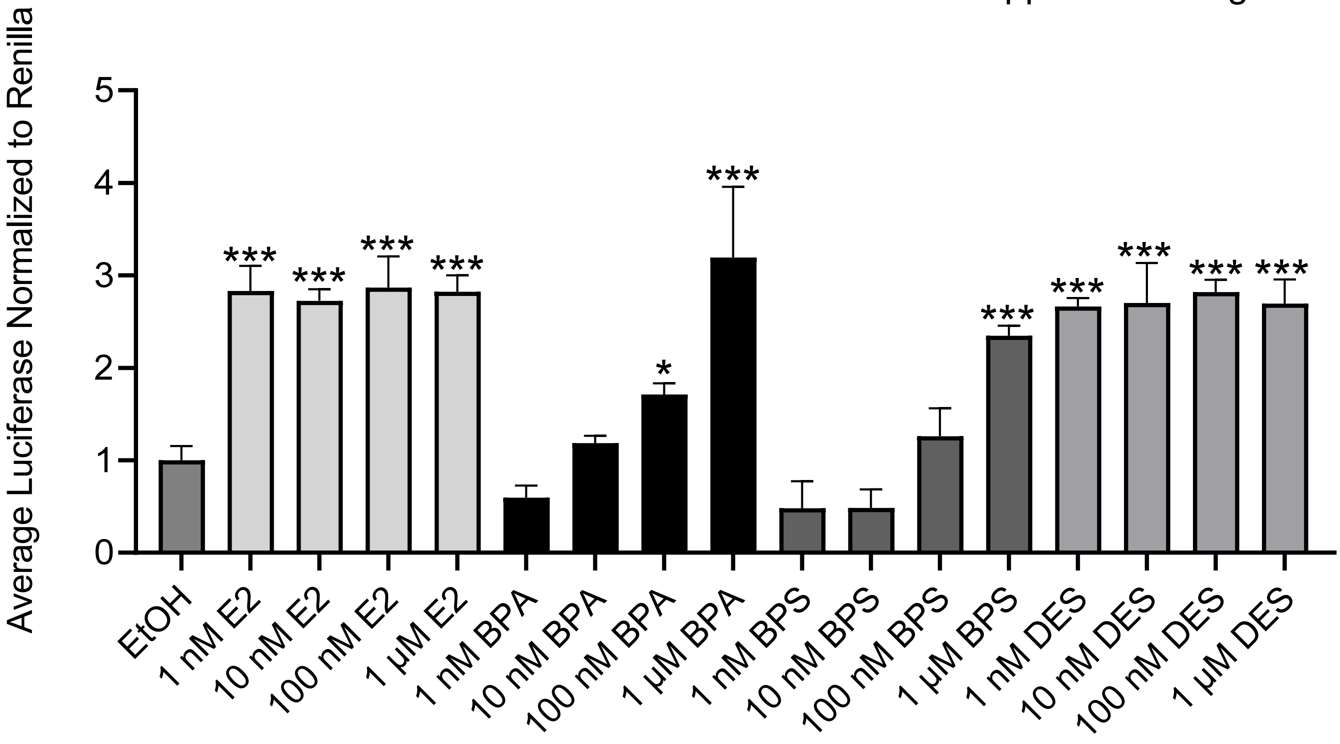
Average Luciferase Normalized to Renilla
0
1
2
3
4
5
***
***
***
***
*
***
***
***
***
***
***
EtOH
1 nM E2
10 nM E2
100 nM E2
1 µM E2
1 nM BPA
10 nM BPA
100 nM BPA
1 µM BPA
1 nM BPS
10 nM BPS
100 nM BPS
1 µM BPS
1 nM DES
10 nM DES
100 nM DES
1 µM DES

A

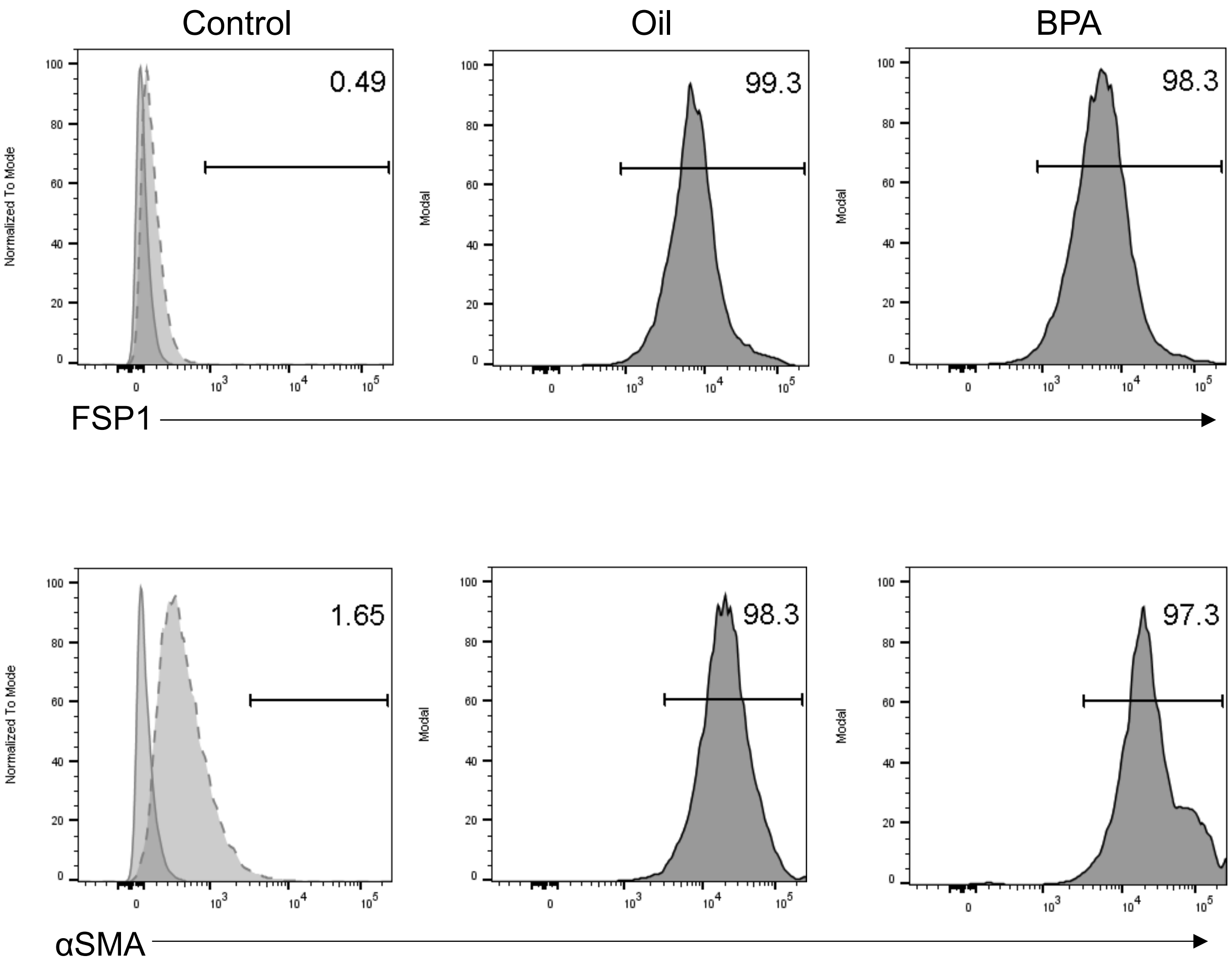
Control
Oil
BPA
0.49
99.3
98.3
1.65
98.3
97.3
Normalized To Mode
Modal
FSP1
αSMA

B

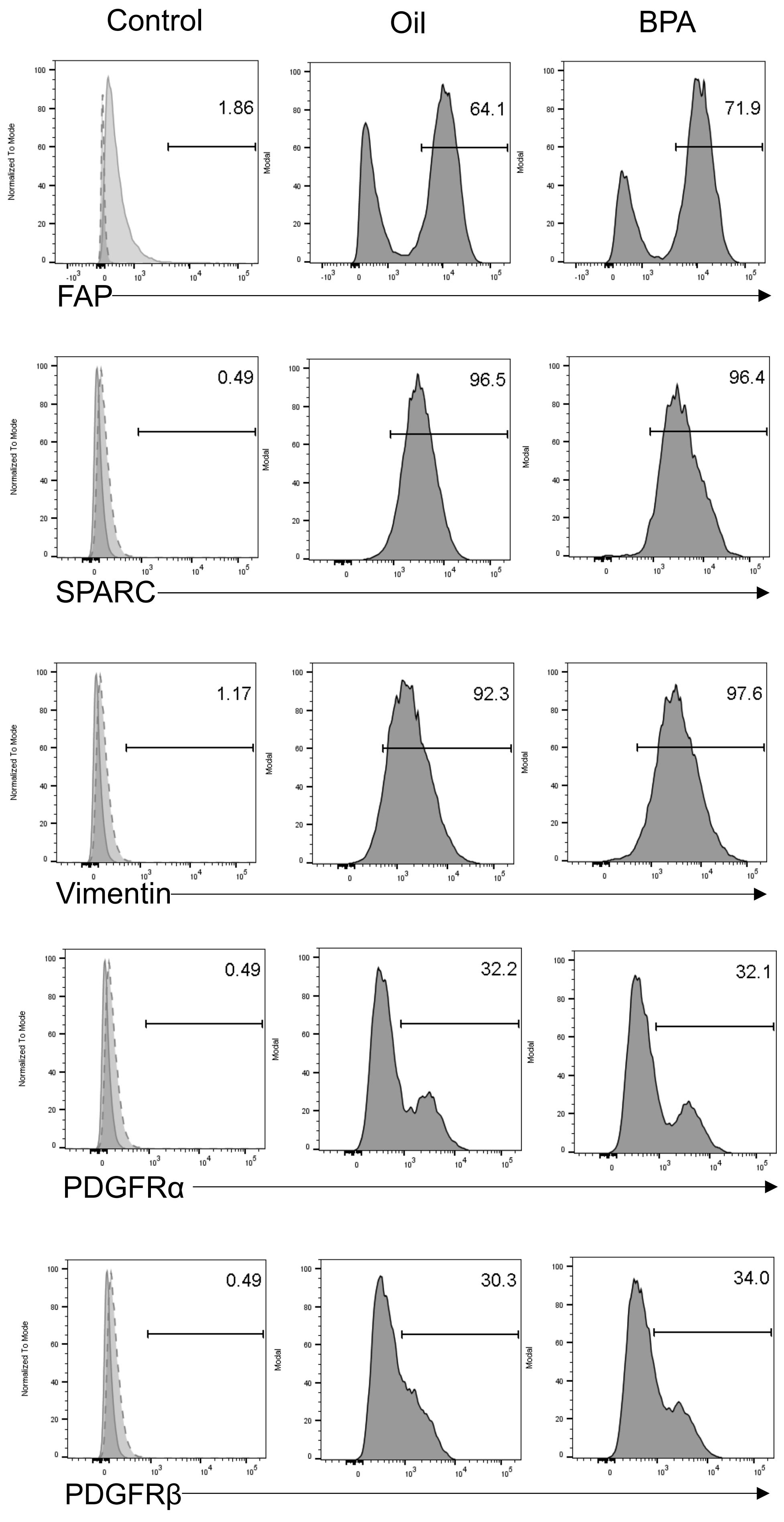
Control
Oil
BPA
1.86
64.1
71.9
FAP
0.49
96.5
96.4
SPARC
1.17
92.3
97.6
Vimentin
0.49
32.2
32.1
PDGFRα
0.49
30.3
34.0
PDGFRβ
Normalized To Mode
Modal

A

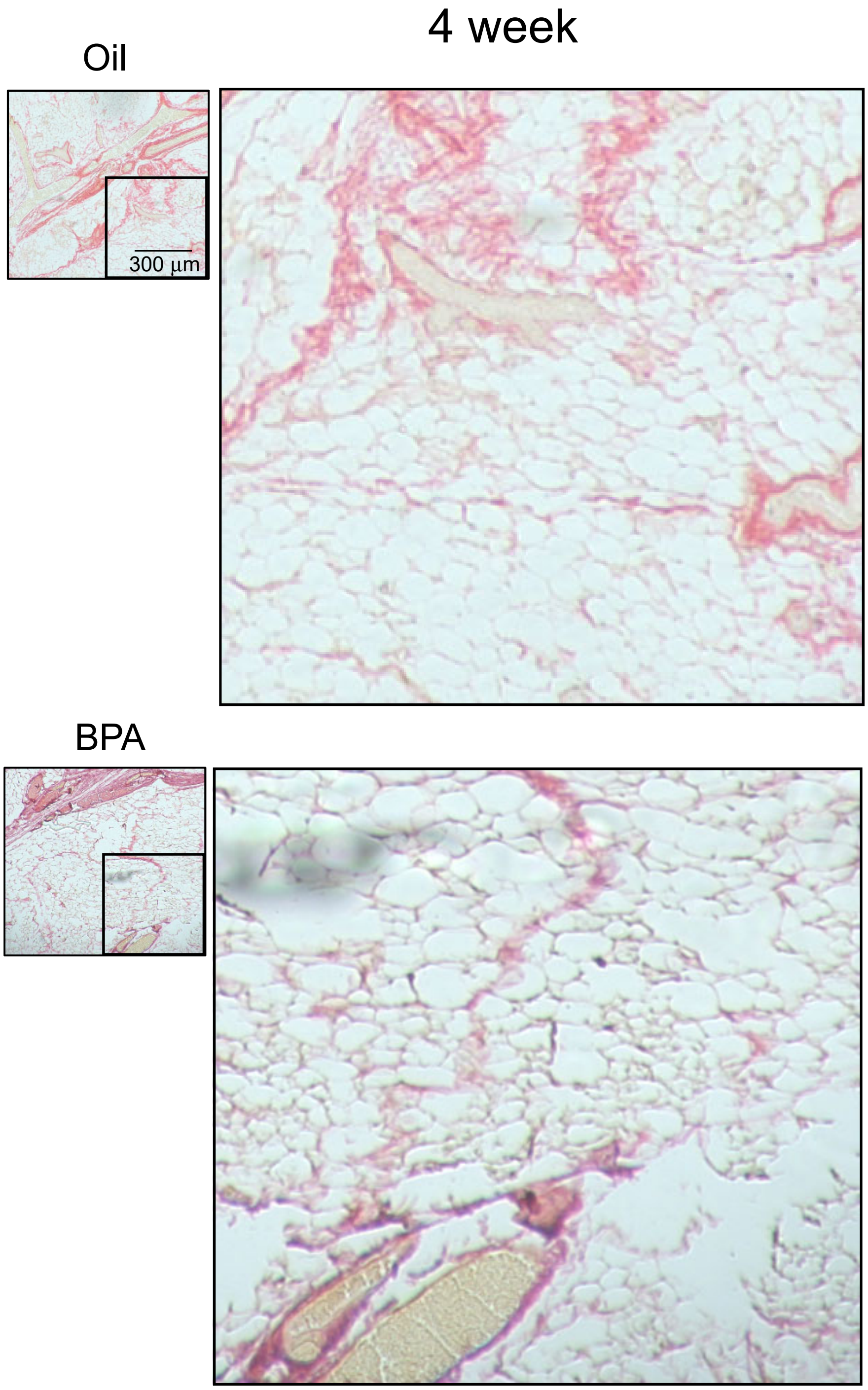

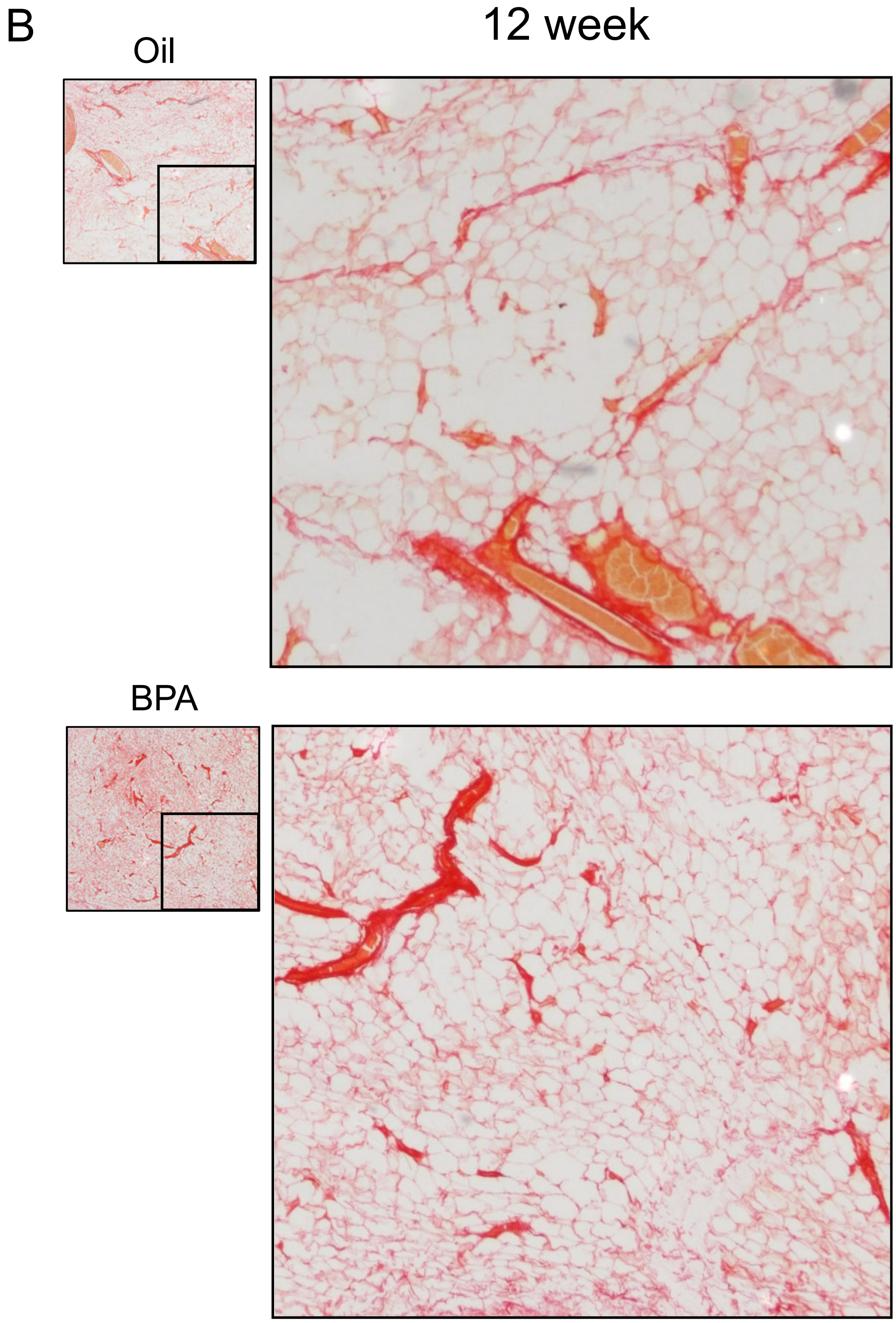
B
12 week
Oil
BPA
